# Bespoke science: the use of ad hoc scientific advisory committees in the Covid-19 pandemic

**DOI:** 10.1007/s11127-025-01299-w

**Published:** 2025-06-19

**Authors:** Roger Koppl, Kira Pronin, Nick Cowen, Marta Podemska-Mikluch, Pablo Paniagua

**Affiliations:** 1 Syracuse University, Syracuse, USA; 2 University of Lincoln, Lincoln, UK; 3 Gustavus Adolphus College, St. Peter, USA; 4 Universidad del Desarrollo, Las Condes, Chile

**Keywords:** Public health, Policy discretion, Policy legitimacy, Experts, Expertise, Scientific advice, Scientific advisory bodies, Scientific advisory committees, Scientific advisory boards, Covid-19, Pandemic, Public choice

## Abstract

Many governments formed ad hoc scientific advisory committees in the Covid-19 pandemic because they offered the government greater control over policy advice than standing agencies. The difference between ad hoc and standing advisory bodies has been little noted in the literature. High-uncertainty crises demanding expertise and requiring action from the national government increase the value of policy discretion, raising the value of controlling the scientific narrative. An ad hoc body is generally easier to control than a standing body because policymakers have greater liberty to choose its members, specify its mandate, and disband and reconstitute it when needed. Control generally requires either a narrow membership or a narrow mandate. If members cannot be chosen to be reliably aligned with the government or its policy preferences, a narrow mandate will restrain the committee from offering undesired advice or analysis. Our argument builds on the public choice assumption of motivational symmetry: The choices of scientists and politicians are shaped by the same motives and desires that influence individuals in any other sphere of life. Our case studies of Italy, UK, US, Poland, Uganda, and Sweden support our theory.

## Introduction

*The scientific advisory committee is a neglected political institution whose importance became clear during the COVID-19 pandemic.*

~ Zeynep Pamuk

Many governments responded to the Covid-19 pandemic by creating ad hoc scientific advisory committees instead of relying on established emergency management and expert structures. What compelled the formation of these temporary entities when the apparatus for crisis management already existed? The gist of our argument is that 1) in crises such as the Covid-19 pandemic, the political motives of policymakers will generally give them a desire to control the scientific narrative, and 2) an ad hoc scientific advisory committee (SAC) is generally easier to control than a standing agency.

While we focus on the recent pandemic, the basic pattern we identify is not new. Nor is it restricted to cases in which the relevant expertise is considered “scientific.” Franklin Delano Roosevelt’s “Brain Trust,” an ad hoc advisory body, is a salient example that provides a template. The Great Depression was a crisis that resembled the Covid-19 pandemic in that: 1) the perceived severity or urgency of the crisis called for action from the national government, 2) there was both unusually high uncertainty about the consequences of alternative policies and ignorance of available policy options, and 3) experts were thought to have superior (if still imperfect and incomplete) knowledge of policy options and consequences. Call such crises “Delanovian” after FDR’s middle name. Notwithstanding the technocratic façade of the Brain Trust, “politics played a central role in shaping the New Deal” (Couch and Shughart 1998, 145). Our argument is that in a Delanovian crisis, including the Covid-19 pandemic, policymakers tend to use ad hoc advisory bodies not only for expertise, but also to preserve their policy discretion, legitimize their policy choices, and to act as a scapegoat should their policies fail. In sum, no crisis, however great, can drive out politics as usual.

Policymakers generally have some desire for discretion: freedom from constraining rules and prior commitments. The value of discretion jumps in a Delanovian crisis because of the high level of uncertainty. You don’t know what the effects of different policies will be, so you need to be able to change course. The available policy options or the situation on the ground may change in unexpected ways, thus inducing a change in desired policy. If politics is exchange (Buchanan and Tullock 1962), then you want to be able to punish non-reciprocating counterparties and, certainly, stop the flow of resources to them. The ability to do so implies policy discretion. New counterparties may arise or you may wish to renegotiate an existing deal, which, again, implies policy discretion.

Our theory builds on the insight that ad hoc advisory bodies assembled for a crisis are easier to control than standing agencies. Policymakers have greater liberty to choose and vary their membership, specify their mandates, and disband and reconstitute them if need be. As long as the decision-maker can find enough competent, yet compliant or controllable experts, they should be able to assemble an ad hoc advisory body that will provide support and cover for the policymaker’s preferred policies even as those preferences change. When the relevant expertise of such a body is scientific, we may call it a “scientific advisory committee” (SAC). Just as tailors produce bespoke suits to fit the needs of their clients, ad hoc SACs produce bespoke science to fit the needs of their creators. Just as a tailor’s suit will hide bodily flaws and improve the client’s appearance, an ad hoc SAC’s scientific analysis and advice will hide political motives and improve its creator’s appearance.

A 1970 Congressional hearing on advisory committees (US House 1970, 46–47) suggests that policymakers understand the strategic use of ad hoc SACs. Testifying on “public advisory groups,” Comptroller General Elmer Staats noted the “danger that the executive agencies will use these groups as crutches. If it is a hot potato or a difficult problem, the tendency is to say, well, we will put it before this group and let them vote in favor or against it.” Representative Monagan responded, that “it might be easier to pass on a decision to a group from the outside than an inter-agency group” and, “You can also determine the composition of the group of people from the outside who might be more sympathetic to your point of view than if you had to pick them out of other agencies of the Government where you wouldn’t have any discretion.”

Recognizing the incentives created by Delanovian crises resolves the puzzle of why so many governments relied on ad hoc bodies to address the Covid-19 pandemic and why such bodies often lacked viewpoint and disciplinary diversity. Viewpoint and disciplinary diversity are inimical to retaining policy discretion and control of the ad hoc SAC. Though the advice of diverse committees may be better by some objective criterion, such committees are less predictable and more likely to become divided, forcing policymakers to choose between competing policy recommendations and exposing political tradeoffs of different policy options. Heterogeneity, then, undercuts the pretense that policy is based strictly on neutral science. External constraints may, however, force policymakers to appoint ad hoc SACs with a diverse membership. In that case, the policymakers will *restrict the committee’s mandate* to secondary issues that present low uncertainty and low risk.

Our investigation illustrates the importance of the “organization of inquiry” (Tullock 1966) in shaping scientific findings used to influence policy decisions (c.f. Leeson, King & Fegley 2020; Leeson 2022; Geloso and March 2021). It challenges the conventional narrative of a seamless transfer of knowledge from scientific advisory committees to governments, underpinned by the belief in scientists’ intrinsic objectivity and governments’ genuine quest for knowledge. Public choice theory, with its principle of motivational symmetry, treats the choices of scientists and politicians as shaped by the same motives and desires that influence individuals in any other sphere of life. We should expect that a government uncompromising in its pursuit of knowledge would foster competition among experts to fortify the credibility of received guidance. The absence of such contestation in the advisory process warrants scrutiny and a search for alternative explanations.

Our paper contributes to the literatures of public choice and political economy in three respects. First, it provides (as far as we know) the first empirical analysis of ad hoc advisory bodies in the Covid-19 pandemic. Other studies have neglected the distinction between ad hoc and permanent bodies. Second, it provides a novel analytical framework to guide studies of crises in which ad hoc advisory bodies are deployed, typically, for political purposes. Third, it provides a new path to develop Tullock’s (1966) research on the organization of inquiry, helping to bring public choice insights to social science studies of the social and natural sciences.

We conducted exploratory case studies of governmental responses to the Covid-19 pandemic in Italy, UK, USA, Poland, Uganda, and Sweden. We found that the most important Covid-19 ad hoc SACs consisted of epidemiologists and related health specialists. There was a notable lack of both stakeholders and experts in diverse fields such as sociology, education, and economics. When political or institutional considerations led to the inclusion of diverse experts and stakeholders, the SAC was constrained by restrictive mandates or political control to prevent unexpected or undesired advice. Even our “deviant” case of Sweden conforms to our theory.

## Literature review

Leeson and Thompson (2023, 6) note that, until recently, “public choice scholars have attended only modestly to issues in public health.” A public choice perspective views both resource allocation and regulation as “often... driven by private interests, not public ones.” It gives due attention to “perverse effects” that thwart the putative goals of public-health measures (p.5). There is now increased attention to such issues (Furton et al. 2023; Kurrild-Klitgaard 2024; Cardella et al. 2024). For instance, Bjørnskov and Voigt (2022a, 2022b) find that governments respond to emergencies by increasing the relative power of the executive, which tends to degrade policy outcomes. Furton (2023) reaches a broadly similar result. Governments respond to public health crises by making structural changes that, in effect, discount long-run consequences, thereby degrading governmental responses to subsequent crises. Leeson and Rouanet (2021) find that private action is more effective in countering “infectious disease externalities” than commonly recognized, in part because property owners can privately regulate or restrict the use of their locations. Recent contributions to this literature include papers addressing aspects of the Covid-19 pandemic. Our paper adds to this literature by examining the strategic role of creating ad hoc scientific advisory committees to advise on (supposed or real) emergencies.

In the political science and policy literatures, ad hoc SACs and their institutional design have received scant attention (Pamuk 2022; Behdinan et al. 2018). The literature on policy advisory systems, which traces to Seymour-Ure (1987) and Halligan (1995), occasionally mentions ad hoc SACs, but generally emphasizes the grand ecology of advising within which governments operate. The strategic and political dimensions of ad hoc SACs are generally neglected (Sato et al. 2014; Morales 2021; Groux et al. 2018). Teller (1988, 346) and Kreidler (1988, 209) uncritically extoll the benefits of ad hoc advisory bodies.

Pamuk’s (2021, 2022) important “paradox of scientific advice” is the tradeoff between utility and neutrality in scientific advice. “Judgments about values and ends” are deviations from scientific neutrality, but necessary for advice to be useful. She argues for the publication of dissenting opinions (2021, 86–87) and for “science courts,” in which citizen juries would judge competing claims of rival groups of scientists. She does not explore the strategic use of SACs, which we emphasize here.

We also build on the blame-avoidance literature initiated by Weaver (1986), who argues that voters are more sensitive to losses than gains (cf. Alesina and Passarelli 2019). Because of this “negativity bias,” politicians prize *avoiding blame* above claiming credit. In this literature “blame” is often *imputation* rather than *imprecation*. Just as a parent may “blame” the doctor for unpleasant medicine their child must take, a government may “blame,” for example, economists for an unpleasant fiscal policy. In both cases, “blame shifting” is possible only because the expert (physician or economist) is viewed favorably. Hood (2002, 2011) says that the inherent risks of policy-making casts policymakers into a “blame game,” which is made more deadly by the news media’s tendency to give disproportionate attention to (supposed) policy failures. Thus, a rational politician adopts blame-avoidance strategies such as bundling budget cuts rather than voting on them separately.

Delegation of policy-making authority is an important blame-avoidance strategy (Weaver 1986; Hood 2002, 2011, Heinkelmann‐Wild et al. 2023). However, it involves a loss of policy discretion (Weaver 1986, 386–387). Hood (2011, 75–76) hints at alternative institutional structures to get around this problem, stating that “top office holders may be able to disavow responsibility while keeping control in practice by behind-the-scenes intervention and arm-twisting.” We develop this insight for the case of ad hoc SACs, which Hood did not consider.

A recent strand of this literature discusses delegation during large-scale, prolonged crises, in which the usual strategy of diffusing responsibility to complex and fuzzy institutional structures (to avoid detection of blame shifting) is infeasible because citizens expect the government to consolidate leadership (Hinterleitner et al. 2023). The government may then turn to experts for blame avoidance purposes. Zahariadis et al. (2020) describe how Turkish and Greek governments depicted experts as policymakers to diffuse accountability for Covid-19 policy. Flinders (2020) suggests that “hugging the experts” lets the government justify decisions and shift responsibility. Hood (2002) cautions, however, that experts can sometimes shift the blame back onto the government. Weaver (2013) briefly mentions delegating responsibility to temporary bodies that are not directly politically accountable as an effective strategy. But there is little attention to the strategic use of ad hoc advisory bodies, which we consider in the following sections.

## Theory

We assume “politicians want to get elected or re-elected” (Buchanan and Tullock 1962, 334). This desire is an important driver of their policy preferences, which include the desire to preserve policy discretion. We have noted that ad hoc expert bodies better preserve discretion because they are more easily controlled. In the cases we consider, the combination of urgency to act and ignorance of consequences increases the value of policy discretion. The relative ease of controlling an ad hoc SAC makes it an attractive option for a government that must act even though it is in alien territory.

In defining a Delanovian crisis we mentioned the “*perceived* severity or urgency of the crisis.” WHO data suggest that Uganda had a much lower Covid death rate than the other countries in our sample. But the forward-looking *perception* of urgency was probably just as great. If a nation’s public has a greater sense of urgency than its government, that nation’s government may be driven to behave as if it shared the public’s sense of doom. A government may also try to induce a perception of urgency in the public, whether cynically or out of a sincere sense of crisis. In any event, it is the perception and not the reality that matters. Thus, we expect (and have seen) broadly similar governmental reactions to Covid from hard-hit Italy and lightly grazed Uganda.

Governments are likely to recognize the risks of broad SAC membership and to minimize them through either a narrow membership or a narrow mandate. Membership may be “narrow” in its political *interests*, or range of *disciplines*. An SAC with aligned political interests is least likely to act in undesired ways but may not provide sufficient legitimacy. Members drawn from a narrow range of scientific disciplines afford more legitimacy at the cost of a greater risk of deviating from the government’s interests. In economics, the existence of different (sometimes quarrelling) schools of thought, such as Keynesianism and monetarism, diminishes the popular prestige of the discipline. This problem is smaller in “science,” which enables governments to make a biased selection with minimal loss of prestige. It is understandable, then, that government actors might wish to represent the Great Barrington Declaration as “fringe” science (Bhattacharya 2023) to tamp down any notion that different, equally legitimate schools of thought had different and inconsistent analyses.

If law, regulation, or political pressure obliges a government to select a broad membership for an ad hoc advisory body, it will constrain the SAC with a narrow mandate. This constraint prevents the SAC from venturing into dangerous topics and giving politically awkward analyses. But it makes the SAC useful for only a relatively narrow range of functions. Thus, there is a tradeoff between viewpoint diversity and the discretion and scope given to scientific advisory committees. Our theoretical considerations lead to the following empirical implications, which (following Eisenhardt 1989) we express as “hypotheses”:

***H1****: In Delanovian crises, policymakers will create ad hoc SACs (charged with evaluating the issue and recommending policies) when: 1) official scientific advice may be expected to enhance policy legitimacy, 2) the policymakers wish to preserve policy discretion, and 3) policymakers anticipate being able to control the advice of the ad hoc SAC.*

H1 reflects our theory that in a Delanovian crisis policymakers tend to use ad hoc SACs not only for advice, but also as a vehicle for politics as usual and that ad hoc SACs tend to serve the political ends of their creators better than standing bodies. An ad hoc SAC may more easily be induced to provide *bespoke science* tailored to the government’s political needs.

***H2****: When policymakers create an ad hoc SAC, they will attempt to control it through a narrow choice of members or through a narrow mandate.*

H2 reflects the trade-off between viewpoint diversity and the discretion and scope given to ad hoc scientific advisory committees. This trade-off exists only because ad hoc SACs serve political ends, whatever their epistemic role may be in individual cases.

## Research design

We conducted case studies of government responses to Covid-19 in the USA, UK, Sweden, Italy, Poland, and Uganda using publicly available primary (government reports, executive orders, etc.) and secondary (published case studies, news, etc.) sources. We focused on the main Covid-19 SACs appointed by national governments and, in one case, the parliament. (See Table 1.)

**Table 1:** Covid-19 ad hoc SACs; membership composition vs. mandate

	**Broad mandate**	**Narrow mandate**
**Narrow membership** (disciplinary narrowness, political alignment or both)	**CTS (Italy)** **SAGE (UK)** **White House Coronavirus Task Force (US)** **White House Covid-19 Response Team (US)** **Crisis Headquarters (Poland) -** **Team for Monitoring and Forecasting the Course of the COVID-19 Epidemic (Poland)** **Medical Council for Covid-19 (Poland)** **Council for Covid-19 (Poland)** **NTS (Uganda)** **SAC (Uganda)**	
**Broad membership** (includes government officials from various sectors, social scientists, stakeholders etc.)		**CES (Italy)** **SBI-P (UK)** **Presidential Covid-19 Health Equity Task** **Parliament’s Covid-19 task force/RTF (Uganda)**

We used Mill’s (1843, 450–454) “method of agreement,” also called the “most-different method” (Gerring 2017, 39–42). In this design, the cases studied “vary widely in background factors regarded as potential causes (Z), while sharing a common outcome (Y).” Factors that “differ across the cases are unlikely to be causes of Y since that outcome is constant across the cases.” But “if a potential causal factor (X) is constant across the cases, it might be the cause of Y” (Gerring 2017, 83–84). Such designs are useful as an exploratory strategy but weaker in terms of causal identification (Gerring 2017, 84). This a limitation of our study. However, social scientists should not abandon their efforts to study a legitimate scientific issue just because a clear causal identification is difficult with the available data (Skarbek 2020).

Our design exposes us to the concern that we may select cases on the dependent variable (formation of ad hoc SACs). However, while our sample was not random, it is not unrepresentative since the majority of national governments formed an ad hoc SAC, and we have Sweden as a prominent counterexample. Unlike the other countries in our study, Sweden *did not* appoint a Covid-19 ad hoc SAC or institute national lockdowns. It is an “outlier,” or “negative” case (Molnar 1967; Emigh 1997). Such cases are useful in finding “a new causal factor, an interaction... effect between two or more causal factors, or a revision to the scope conditions of the theory” (Gerring 2017, 75).

Greer et al. (2020, 1413) propose that governmental COVID-19 responses could be explained by “social policies to crisis management as well as recovery, regime type (democracy or autocracy), formal political institutions (federalism, presidentialism), and state capacity.” Our chosen cases exhibit variation in these Z factors, though we exclude the first category of factors because the crisis management and recovery policies were typically chosen *after* the appointment of a Covid-19 ad hoc SAC. And, importantly, all the countries in our sample met the conditions of a Delanovian crisis given above. Our cases vary also in the initial arrival and severity of the pandemic (Italy reached a Covid death rate of 1 per million before the others. Italy and, especially, Poland rose rapidly to that dreadful benchmark after recording their first confirmed cases. Uganda had a low case count and late arrival of the pandemic). Early arrival and initial severity of Covid-19 might be thought to compel a government to appoint ad hoc SACs, making it a likely Z factor. Our sample also exhibits geopolitical variation, though we did not consider it a potential causative factor, except through the other Z factors.

Regime type is reflected in the 2020 values of the V-Dem (Varieties of Democracy Project) presidentialism index (v2xnp_pres), which measures the de facto level of the centralization of political power. Countries high on this score (Poland and Uganda) might have fewer constraints in appointing ad hoc SACs, making it another Z factor to consider. As a robustness check, we used the 2020 values of V-Dem Rigorous and Impartial Public Administration Index, which captures “the extent to which public officials respect the law and administer it without arbitrariness and bias.” To measure administrative capacity, we used the 2020 World Bank’s WGI government effectiveness rating (Kaufmann et al. 2003). Countries with the lower administrative capacity (Poland and Uganda) might be more likely to appoint ad hoc SACs and countries with especially high administrative capacity (UK, USA, and Sweden) might be less likely to do so. Finally, we noted whether the country was a federal (USA) or unitary state, because one might expect federated states to be less likely to have a centralized response. (But see Hegele and Schnabel 2021.)

## Results and case narratives

### Hypothesis 1

The conditions of H1 were fulfilled in Italy, US, UK, and Uganda. Each created an ad hoc SAC as the government’s main policy advisor.

***Italy:*** In Italy, the Department of Civil Protection issued an ordinance on 3 February 2020 (n. 630) creating the *Comitato Tecnico-Scientifico* (CTS) or “*Technical and Scientific Committee*,” which was Italy’s “main advisory body” on Covid-19, at least through the first wave of the pandemic (Camporesi et al. 2022).

***United Kingdom:*** In the UK, the national government’s main advisory body was the Scientific Advisory Group for Emergencies (SAGE). The UK has a formal provision for the “activation” of a SAGE in an emergency, and the resulting body is an ad hoc SAC. Koppl (2023, 110–111) describes the administrative procedures surrounding the activation of a SAGE and notes, “The government calls each meeting separately and the list of participants varies from meeting to meeting (Government Office for Science 2021).”

***United States:*** In the US, the main Covid advisory body was initially the *White House Coronavirus Task Force*, which President Trump created on 29 January 2020. President Biden replaced it with the *White House Covid-19 Response Team* (Executive Order 13987) immediately after he was sworn in on 20 January 2021. At the same time, he also created the *Presidential Covid-19 Health Equity Task Force* (Executive Order 13995).

***Uganda:*** In Uganda, there were two main advisory bodies, both ad hoc. In June 2020, the Ugandan government created the *National Task Force* (NTF), which was run through the Office of the Prime Minister and “comprised political and technical leaders from the most influential government entities, including Health, Education, Trade, Finance, Tourism, Public Service, and joint security agencies” (Bukenya et al. 2022, 17). Around the same time, “The minister of health invited eminent scientists in the country to form the SAC [*Scientific Advisory Committee*] to lead research, innovation and generate scientific evidence to advise MoH [Minister of Health] and NTF” (Bukenya et al. 2022, 19).

The conditions of H1 were not fulfilled in Sweden or, initially, Poland.

***Sweden:*** In Sweden, condition 2), the government’s desiring policy discretion, did not hold. This surprising state was brought about by the 2019 “January Agreement” (*Januariavtalet*), which bound the government closely to a rigid policy program. As we discuss below, the ruling coalition had to agree to a stringent policy program to get enough votes in the Swedish parliament to come into power. Crudely, the government “in power” had no power and, therefore, could not exercise policy discretion.

***Poland:*** In Poland, condition 3), ability to control the SAC, did not hold until well into the pandemic. Early in 2020, Poland’s ruling Law and Justice party, anticipating victory in the May 10 presidential election, refrained from declaring a state of emergency that would have delayed the vote and jeopardized its electoral advantage. The government likewise avoided forming an ad hoc scientific advisory committee that might recommend postponing the election or tightening lockdowns—steps that could harm the party’s interests.

Only in November 2020, with the election concluded and a second wave underway, did the Prime Minister create an ad hoc Medical Council for Covid-19 (Rada Medyczna). This new body legitimized strict lockdown measures and helped address widespread protests. However, its advice, especially on vaccination policy, soon clashed with government priorities. In January 2022, multiple members resigned over official tolerance of anti-vaccine rhetoric, prompting the government to disband the Council. It was immediately replaced by the Council for Covid-19 (Rada ds. Covid-19).

Policymakers delayed creating an ad hoc SAC until they could safeguard their political objectives (Condition 3). Once all three conditions of Hypothesis 1 were met, the government successively formed and replaced ad hoc SACs to manage Covid-19 policy on its own terms.

### Hypothesis 2

H2 was not contradicted by our data. Table 1 classifies the Covid-19 ad hoc SACs in our sample by their membership composition and mandate. Consistent with H2, highly representative SACs that were not identifiably aligned with the government had the most restrictive mandates.

***Italy:*** In Italy, the CTS had a “narrow range of expertise” and was highly aligned with the government (Pistoi 2021). Camporesi et al. (2022) note a “near-complete overlap of technical advice and political response in the first phase of the pandemic in Spring 2020, with a key role played by the advice provided by the Technical and Scientific Committee (CTS).” The ordinance creating the CTS (Ordinanza del 03/02/2020 n. 630) gives it a broad mandate that does not restrict the topics the CTS may address.^1^ Its preamble even speaks of the “extraordinary means and powers to be employed” in the emergency. As we discuss below, the more broadly representative ad hoc Committee of Experts in Economic and Social Matters (CES) was also created by decree, but its mandate limited it to measures to aid recovery after lockdown.

***United Kingdom:*** In the UK, the SAGE membership was narrowly drawn from epidemiology, public health, and other medical specialties, with no socioeconomic or other non-medical experts, though a few academic statisticians and biologists were included. SAGE had a broad mandate. Its “guidance document” (SAGE n.d.) explicitly says it is “flexible” and its “precise role will evolve as the emergency develops.” The more broadly representative SBI-B, the behavioral subcommittee of the SAGE, had a narrow mandate that mostly restricted it to designing behavioral “nudges” to implement SAGE’s policy recommendations (Koppl 2023, 116).

**Fig. 1:**
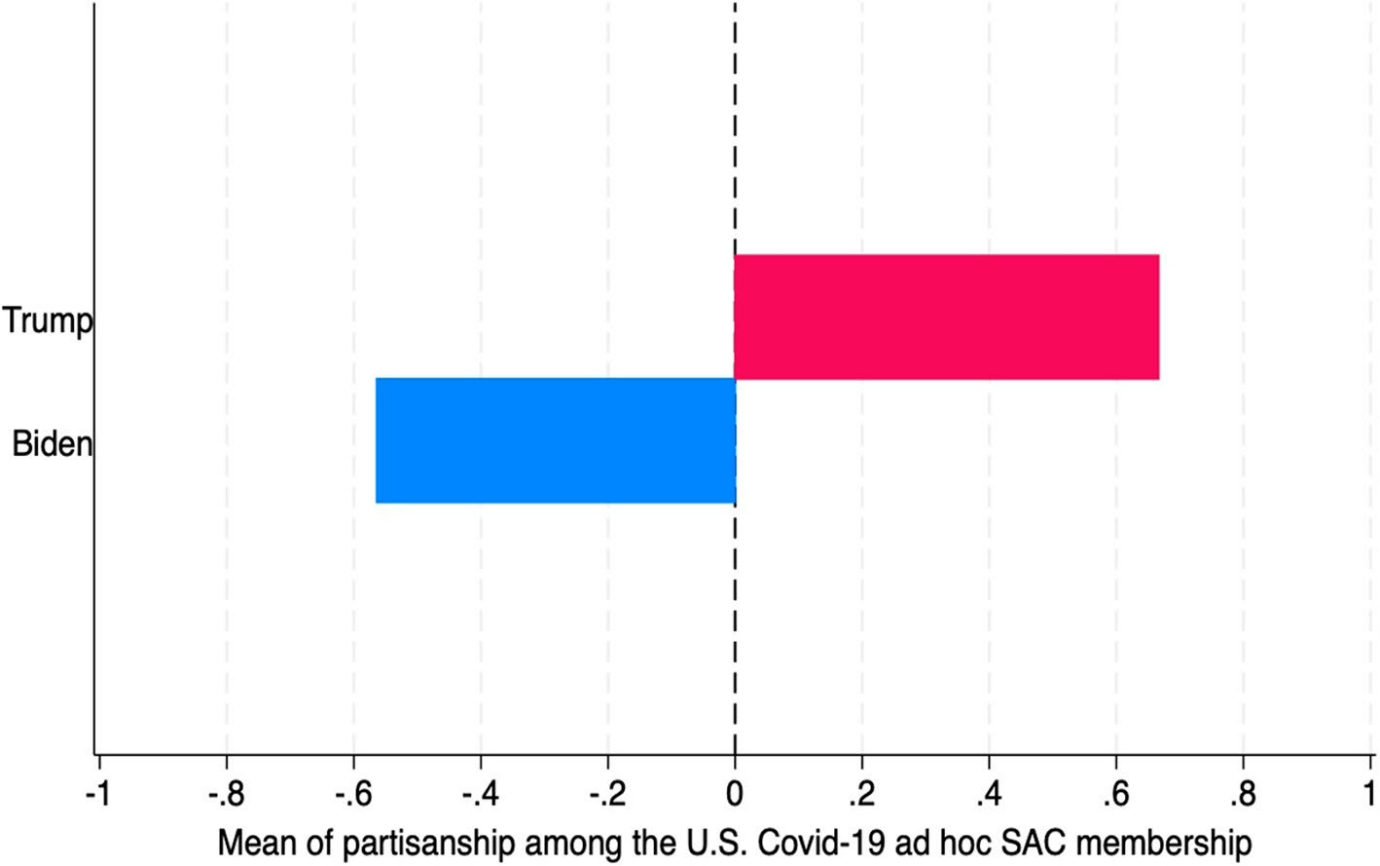
**The Blue Shift accompanying the change of administration.** *Note* We searched public sources for party affiliations, scoring members as -1 if they were clearly Democrats, + 1 if Republicans and zero otherwise

***United States:*** In the US, narrow membership was achieved through political alignment. Both Trump and Biden appointed *politically aligned members*, which led to the “blue shift” represented in Fig. 1. Both bodies had broad mandates. Trump’s Task Force was to “lead the Administration’s efforts” and Biden’s Response Team was to “coordinate all elements of the COVID–19 response.” Biden’s *Presidential Covid-19 Health Equity Task Force* had a broad membership, which was constrained, however, by a narrow mandate.

***Uganda:*** In Uganda as well, narrow membership was achieved through political alignment. The Ugandan SAC and NTF were both highly aligned with President Museveni who put his friends on them (Mbonye 2020).

***Poland:*** In Poland, narrow membership for the *Medical Council for Covid-19* took the form of disciplinary narrowness. Its members were experts in epidemiology and infectious diseases. Narrow membership for its replacement body, the Council for Covid-19 was achieved through political alignment.

### Case 1: Italy

Consistently with H2, membership of CTS, the main advisory body during the pandemic, was narrowly drawn with no socioeconomic or other non-medical experts. Camporesi et al. (2022, 7) report, “the expert advice that supported the management of the pandemic in Italy saw a strong predominance of epidemiologists and infectious disease experts. The input of socioeconomic experts, ethicists and other non-health disciplines did not have a substantial impact on policy decisions.” Pistoi (2021) similarly notes “a predominance of epidemiologists and infectious disease specialists over social scientists in the mobilization of experts for the management of the crisis in Italy”.

Consistently with H2, the broadly representative CES, created by a decree of Prime Minister Conte on 10 April 2020, was constrained in ways that preserved the government’s policy discretion. It was charged with “developing and proposing... measures necessary to cope with” the emergency “as well as” devising measures for “the gradual resumption of the diverse sectors of social, economic, and productive activity” in part through the “identification of new organizational and relational models that take into account the emergency needs of containment and prevention” (Presidente del Consiglio 2020). While the initial bit of the mandate seems broad and open ended, the Prime Minister’s decree requires the CES to act “in coordination with” CTS, which blunts its independent authority. And the more specific bits suggest a narrower function for the committee. It was not the job of the CES to judge non-pharmaceutical interventions such as lockdowns, but to “provide key recommendations on how to support the Italian social and economic recovery *after* the lockdown” (Camporesi et al. 2022, emphasis added).

Political influence was applied to Italy’s CTS. Deputy Minister of Health Pierpaolo Sileri lamented, “CTS is at the service of politics, not the reverse.” (unattributed 2020). We have quoted Camporesi et al. (2022) on the CTS’s “near-complete overlap of technical advice and political response.” This “overlap” is reflected in a story Pistoi (2021) tells. At the peak of the crisis in March 2020, “a group of 292 Italian scientists” volunteered their labs and personnel for Covid testing. CTS rejected their offer on the dubious grounds that research labs were not the best place to perform diagnostic tests and, in any event, Italy was ramping up its diagnostic capacity. We wonder if they feared losing control of the narrative if they let other scientists into the tent. Whatever CTS’s true motive may have been, its choice seems more political than scientific. It was, Pistoi (2021) claims, “the first of several decisions taken by CTS that, over the past year, have puzzled Italian experts who view testing and tracing as key.” The alignment (“overlap”) of CTS with the reigning political interests of the Italian state is consistent with our view of the role of ad hoc SACs. They give scientific cover to the government’s preferred policies.

### Case 2: UK

The British government relied mostly on the (ad hoc) *Scientific Advisory Group for Emergencies* (SAGE) for policy advice. SAGE membership was narrowly drawn from epidemiology, public health, and other medical specialties, with no socioeconomic or other non-medical experts, though a few statisticians and biologists were included. Koppl (2023, 114–115) discusses a guidance document in which “Science and technical advice” is distinguished from “Economic advice” and “Legal advice.”

SAGE’s behavioral science subcommittee, the Scientific Pandemic Insights Group on Behaviours (SPI-B), adds disciplinary breadth to SAGE. However, per the UK’s Government Office for Science (2021), SBI-B “provides advice aimed at anticipating and helping people adhere to interventions that are recommended by medical or epidemiological experts.” It was, again, designing nudges, not policy.

The government also applied political pressure to SAGE. Its meetings often included “attendees” to “provide the scientific experts with context on the work of government where appropriate.”^2^ Beginning with SAGE9, which met on 20 February 2020, meetings regularly included personnel from No 10. Freedman (2020, 515) argues, “This raised the question of whether the process was being manipulated so that the only advice received fitted with the government’s established preferences.” He notes the “risks in politicising scientific advice, of the sort that were feared when it was reported that Dominic Cummings [a close advisor to PM Boris Johnson] had attended SAGE meetings” (Freedman 2020, 521). SAGE minutes for 27 February reveal SAGE’s consensus view that it would be better to impose relatively severe restrictions and slowly relax them than to impose more modest measures at first and slowly ramp them up. Freedman laments, “SAGE ended up pushing the second option.” He infers, “This meant that SAGE was taking what had been described previously as an essentially political position” (2020, 519).

Kettell and Kerr (2022, 27) find the UK government hiding behind science when crafting policy. They describe a “constant shifting of the narrative” as ministers were “forced to steer between rapidly moving responsibility in such a way as to minimize blame and maximize credit for their actions”. Salient among their “narratives” was that of “scientific guidance,” which “was evident from the very first briefings.” They quote PM Johnson insisting that his government was acting “not according to political diktat, but according to the best scientific and medical advice in the world” (Kettell and Kerr 2022, 26).

### Case 3: United States

Both the Trump and Biden administrations appointed Covid-19 ad hoc SACs. Trump’s *White House Coronavirus Task Force* consisted of a chair, a Covid-19 coordinator (Deborah Birx) reporting to the President, medical experts, personnel from OMB, the Domestic Policy Council, Homeland Security, the Department of Transportation, and other offices (White House Press Secretary 2020). In May 2020, members with expertise in vaccines, therapeutics and worker safety were added. Biden’s *White House COVID-19 Response Team* consisted of a Covid–19 Response Coordinator (Jeffrey Zients), a Deputy Coordinator (Natalie Quillian) and 13 members, including medical professionals, government officials and experts on data and communication, but lacked socioeconomic experts (Zraick 2021). Six members had advanced degrees from Harvard, Yale, and Columbia Universities. The educational backgrounds of others varied.

Trump’s more broadly representative Task Force was subject to greater political control. After an initial period under Alex Azar of Health and Human Services, Vice President Mike Pence was put in charge of the Task Force. Consistently with H2, Biden’s *Presidential Covid-19 Health Equity Task Force*, which was broadly representative of various stakeholder interests, at least on paper, was constrained by a mandate to “provide specific recommendations” for “mitigating” “health inequities” with an emphasis on the governmental allocation of “COVID–19 resources,” not issues related to the overall pandemic strategy.

Consistently with H2, the members of the Task Force and Response Team appeared to have been selected on partisan grounds (see Fig. 1). About 67% of Trump’s Task Force was identifiably Republican, and none were identifiably Democrat. About 56% of Biden’s Response Team was identifiably Democrat, and none was identifiably Republican. This “blue shift” strengthens the view that their memberships were chosen for political ends to steer the scientific decisions reached within the committee.

The Response Team and Task Force were unusual in that their members were *government employees*. Such members were likely chosen to circumvent the Federal Advisory Committee Act (FACA) of 1972, which requires that advisory committees be “fairly balanced in terms of the points of view represented and the functions to be performed.” (More recently, Trump’s motive for creating DOGE through the “reorganization and renaming of the United States Digital Service” was probably to escape FACA restrictions. See Elliott 2025.) FACA also requires that (subject to a reasonability requirement) “Interested persons shall be permitted to attend, appear before, or file statements with any advisory committee.” These and other provisions of FACA make Federal Advisory Committees ill-suited for blame avoidance and discretion preservation. An essential obstacle to political uses of ad hoc SACs is FACA’s broad definition of “Federal Advisory Committee.” The definition stipulates that any strictly advisory “committee, board, commission, council, conference, panel, task force, or other similar group, or any subcommittee or other subgroup thereof” counts, *excluding only bodies composed exclusively of federal employees* or created by National Academy of Sciences or the National Academy of Public Administration, though there are carve-outs for national security, intelligence, and the Federal Reserve System.

Trump’s Task Force did not seem to deliver the recommendations he was looking for. On 5 May 2020, he confirmed his intention to “wind down” the Task Force and establish a “different group” to “open our country” (Trump 2020a). On 6 May he reversed this decision in response to a backlash. “I had no idea how popular the task force is until actually yesterday” (Trump 2020b). He did, however, add new members and change the Task Force’s mandate to focus on “getting Americans back to work and allowing businesses to re-open.” (Trump 2020b). By the end of May the Task Force had reduced its meetings from daily to once per week. It existed in this rump form until the end of Trump’s presidential term, having its importance and mandate diminished in what we might call “constructive disbanding”. Birx, the coordinator of the Task Force, later complained that the group had been under-staffed and that “…she felt her science-based guidance was being censored by the White House and that she was being deliberately blocked from appearing on national media outlets for a time” (Quinn and Brennan 2021).

Biden’s executive order made the White House Covid-19 Response Team responsible for “coordinating” various Covid-fighting “efforts” of the Federal Government. This made the Response Team more than advisory, which put it beyond the reach of FACA. Moreover, the members were made federal employees. For example, when Bechara Choucair was brought over from Kaiser Permanente he was made “White House COVID-19 Vaccinations Coordinator,” a full-time White House position (Diamond 2020).

### Case 4: Poland

The Covid-19 pandemic reached Poland on the eve of a highly contentious presidential election scheduled for May 10, 2020. For the ruling Law and Justice party (PiS), the election presented an opportunity to consolidate its hold on power. For the opposition, it was a chance to disrupt the long-held monopoly of the Law and Justice party. The pandemic put into question whether it was safe to hold elections (Meyer 2020; Sagan et al. 2022). With President Andrzej Duda, a Law and Justice loyalist, leading in the polls, the ruling party insisted on holding the elections as scheduled. The opposition called for postponement, expecting that pandemic challenges would decrease Duda’s lead (Badora-Musiał and Dusza 2021). The ensuing political battle shaped Poland’s early response to the pandemic, and likely explains the government’s initial reluctance to appoint an ad hoc SAC (Styczyńska and Zubek 2023).

Poland’s constitutional provisions for states of emergency (including epidemics) require delaying the election for at least 90 days after the termination of emergency measures, which would have put PiS at a disadvantage. Instead, while acknowledging the severity of Covid-19 (Meyer 2020; Sagan et al. 2022), the government circumvented these provisions by passing a Special Covid-19 Act on March 2, 2020.^3^ The opposition deemed this move unconstitutional and argued that the Act introduced a state of emergency “through a kitchen door.”^4^

A Covid-19 medical expert committee would probably have advised postponing the election. At the time, expert consensus favored stringent virus-containing measures and medical experts would have refused to endorse in-person voting. This clashed with the government’s political timetable. As late as in April, even the Law and Justice Minister of Health, Łukasz Szumowski—a cardiologist —resisted endorsing in-person voting and recommended mail-in voting as a safer option. Under political pressure, Szumowski later downplayed the epidemiological risks, aligning his public stance more closely with the government’s interests (Badora-Musiał and Dusza 2021). Ultimately, after significant conflict, the election was postponed to June 28, with a potential second round on July 12 if no candidate secured an outright majority. In the lead-up to the vote, the government’s Covid-19 response paralleled that of other European countries (Krakovsky 2020; Sagan et al. 2022).^5^ As the election got closer, stringent lockdown measures were rapidly lifted. By mid-June, Poland had fewer restrictions than Sweden (Hale et al. 2021).

In Fall 2020, Poland experienced a severe second wave of the pandemic, which overwhelmed its healthcare system and strained critical resources. In early November 2020, the excess mortality rate hit 108 percent—the highest among all EU countries at that time (Mathieu et al. 2020). This crisis coincided with the eruption of massive protests known as the “Women’s Strike” (Strajk Kobiet). Sparked in late October 2020 by the Constitutional Tribunal’s decision to tighten Poland’s already restrictive abortion laws, the protests brought tens of thousands into the streets. Proposals to introduce more restrictive sex education laws further inflamed public anger and fueled the demonstrations. Against this backdrop, imposing a stricter lockdown aligned both with expert medical advice and the government’s strategic interests. On the one hand, health experts naturally advocated renewed or extended lockdown measures to contain the virus’s spread. On the other, enhanced lockdown restrictions would also discourage mass gatherings and dampen protest activity.

With the altered political situation, and consistent with H1, official scientific advice could now enhance policy legitimacy. As we noted above, in November of 2020 the Prime Minister created the *Medical Council for Covid-19* (*Rada Medyczna*), which became the government’s main Covid-19 advisory body.^6^ The Medical Council comprised 15 (later 17) experts in epidemiology and infectious diseases. It was created at the height of Poland’s second wave of the pandemic, when the government faced mounting pressure to address the rapid growth in cases. However, the Council’s influence and independence were constrained by its design: members were appointed and could be dismissed at the Prime Minister’s discretion, ensuring that its advice was not binding in any way.

The Council issued only two formal recommendations in November 2020, after which it remained silent until February 2021. This long silence raises questions about the Council’s role in shaping the National Vaccination Program, unveiled by the government on December 15, 2020. While the Medical Council was listed as one of four entities responsible for developing vaccination priorities, its actual influence remains uncertain. Meetings were not recorded, and some changes to vaccination schedules appeared to originate with the government rather than the Council. For instance, on March 5, 2021, the Council recommended extending the interval between AstraZeneca vaccine doses to 12 weeks and Pfizer doses to 42 days. However, this change had already been announced publicly by the Chief of Chancellery, Michał Dworczyk, a day earlier, highlighting the Council’s reactive role in formalizing decisions.

The Council’s narrow membership, primarily composed of medical experts, created alignment on pushing for vaccination policies, which came to conflict with the government’s political priorities. By the summer of 2021, this friction became visible. When the Council began advocating for a third vaccine dose, Minister of Health Adam Niedzielski publicly cast doubt on the necessity, stating on August 5, 2021, *“I'm trying to balance how much of it is added value for public health and how much of it is the interest of pharmaceutical companies who want to sell more.”* Niedzielski’s comments offended the medical community and contributed to growing vaccine hesitancy, even though the Council did not formally recommend boosters until mid-September.

In January 2022, a conflict erupted over comments made by Barbara Nowak, the educational curator of Lesser Poland, who referred to Covid-19 vaccines as an “experiment.” The Medical Council demanded Nowak’s dismissal, issuing a strong statement defending vaccinations and condemning public officials who undermined trust in the vaccination program. Despite this, the government refused to dismiss Nowak, reflecting its reluctance to act against political allies. Thirteen of the Council’s 17 members resigned in protest, citing the government’s tolerance of anti-vaccine rhetoric and disregard for their advice. The Medical Council was subsequently disbanded on January 21, 2022.

On the same day, the Prime Minister established the *Council for Covid-19* (Rada ds. Covid-19), a new advisory body with a broadened membership of 29 persons, including medical experts, economists, institutional representatives, and socio-economic advisors.^7^ While this broader membership appeared to signal a shift in strategy, the institutional design remained identical: the Prime Minister retained full control over appointments and dismissals, ensuring continued political control and, therefore, policy discretion. In effect, the government resolved the earlier problem of disciplinary alignment—where medical experts pushed for scientifically-driven policies like vaccinations—by creating a more heterogeneous body that diluted the influence of medical voices. The Prime Minister explained that the inclusion of non-medical experts was intended to balance “medical reasons, health care reasons, which have a common denominator called truth” with “expectations, which have a common denominator of a nature, or under the name of freedom.”^8^ Though representing a broader set of disciplines, this group's membership was also narrow because members were chosen to be politically aligned with the Prime Minister’s party (Law and Justice), just as in the case of the Medical Council.

### Case 5: Uganda

In June 2020, the Ugandan government responded to the Covid-19 pandemic by establishing the *National Task Force* (NTF), which was operated through the Office of the Prime Minister, and the *Scientific Advisory Committee* (SAC). The scientists of the SAC were drawn from a wide variety of disciplines including veterinary medicine and psychology (ibid. p. 19). Also in June 2020, the Ugandan parliament created the 40-member *Parliament’s Covid-19 task force* consisting of MPs from various parties and regions. The task force represented the parliament to the NTF and coordinated the interventions of the four *regional parliamentary task forces* (RTFs) announced the same day.

Although the members of the NTF and SAC represented a broad variety of fields, they were reliably aligned with the government through personal ties to the President. A former Director General of Health Services in Uganda’s Ministry of Health has lamented, “Hurriedly, the government assembled a national task force [NTS] chaired by the Prime Minister and a scientific committee [SAC] was also assembled by the Health Minister. Both committees did not have eminent people who had training in infectious diseases nor experience in managing epidemics. Instead, the committees were staffed by acquittances [*sic*] and friends of some sort of kind” (Mbonye 2020). As if to confirm this charge, President Museveni called the SAC “my scientists” (Mbonye 2020, 20).

Consistently with H2, the regional task forces, which had broad party/regional representation by design, had no power to make or advise on core Covid-19 policies. Their mission included assessing the operation, administration and management of their funds and other resources. They were also expected to carry out field visits and assess the state of health care systems in regional referral hospitals and districts, to regularly brief the Speaker and the Parliament’s Task Force and to submit a report to parliament for debate. The Task Force was essentially an intermediary between the NTS and the RTFs. It thus represented no threat to the government’s policy discretion.

Uganda has been praised for its “sustained” success against Covid (Sachs et al. 2020), its speedy move to “strict lockdown” (Biryabarema 2020), and for “listening to science” and “empirical evidence” (Ajuna 2021). But Bukenya et al. (2022, 20) interviewed members of Uganda’s Scientific Advisory Committee and its NTF and found that “Covid-19 policy advice was not purely premised on scientific evidence”. One member of the Committee noted that many “considerations” could come between scientific opinions and the policy that finally emerged: “And then the Cabinet injects its realities” (Bukenya et al. 2022, 21). Another noted that lobbyists influenced Uganda’s Covid policy and the Committee’s advice: “Some groups had bodies/organizations, like the traders, they have KACITA [Kampala City Traders Association] who would write to the committee that they needed their business and trading sector opened. They would give reasons why it should be opened. And then we would give the scientific perspective of opening that sector” (p. 21). Bukenya et al. (2022, 21) say, “Another respondent from the NTF observed that those sectors with well-organized groups were able to have their activities opened, while the less organized ones remained closed.” Laing et al. (2024) argue that government action had “little or no effect” on the course of the pandemic.

The Ugandan executive also used Covid-19 restrictions as a cover for political oppression. NTS and SAC recommendations did not directly call for political repression, but they enabled it. Grasse et al. (2021, 132) describe Uganda’s Covid policies as “opportunistic repression”. For example, opposition leader Bobi Wine “was physically blocked by the army when trying to enter a radio station” (Grasse et al. 2021, 156). “Museveni banned food distributions— a common electoral strategy—following the COVID-19 lockdown,” but enforced the ban only on his political enemies. The “independent MP Francis Zaake” was arrested for distributing food and “tortured while in police custody.” The authorities practiced “forbearance,” however, when members of the President’s political party distributed food (Grasse et al. 2021, 156–157). The regime grew more oppressive over time, with “the increase in repression concentrated in opposition areas that showed less support for Museveni in the 2016 election” (Grasse et al. 2021, 132). Parker et al. (2022) find that “forms of public authority have been enhanced [in Uganda] that reinforce unaccountable governance, link disease control with violent abuse, and are resisted in ways that are unlikely to be associated with improved health outcomes.” Importantly, oppressive policy actions were often aimed at serving President Museveni’s political interests. In one local market, soldiers descended “within hours” of the imposition of the first lockdown” which “was characterized by extensive violence” (Ibid.). The soldiers “fired live rounds of ammunition into the air and beat traders until they ran away – although few people knew that trading was prohibited” (Ibid.).

### Deviant case analysis: Sweden

In contrast to many countries, Sweden relied on its *existing institutions* in setting pandemic policy. The most important were the Public Health Agency of Sweden (*PHAS*) with state epidemiologist Anders Tegnell playing a key role in shaping the Covid-19 response strategy, and the National Board of Health and Welfare (*Socialstyrelsen*), which ensured health care capacity and issued recommendations about various topics, such as end-of-life-care for Covid-19 patients (Olofsson and Vilhelmsson 2022). Sweden’s 21 regional councils (including 21 county administrative boards) and 290 municipalities were also key players in the pandemic response, planning and implementing the local responses in light of the national recommendations (Tegnell 2021).

The Swedish government did not declare a state of national emergency or implement lockdowns, mask mandates, daycare or primary school closures, or stay-at-home orders during the pandemic (Kavaliunas et al. 2020; Nilson 2021; Pierre 2020). Instead, citizens learned about voluntary self-protection measures from the PHAS website and frequent press conferences held by Tegnell and Prime Minister Stefan Löfven (Giritli Nygren and Olofsson 2020). Sweden’s Covid policy was not strictly hands-off, however. In 2020 the *Riksdag* introduced several legally binding regulations with limits on public gatherings, restaurant operations, and visits to elder care. In January 2021, the provisional Covid-19 Act [2021] added a series of further restrictions with concomitant enforcement instruments, which expired in September 2021 (Lynggaard et al. 2023). Nevertheless, the restrictions remained minimal.

Researchers have attributed Sweden’s unusual policy path to constitutional blocks to ministerial interference with public agencies and implementing restrictions of movement except at time of war, as well as to high public trust (Bylund and Packard 2021; Jonung 2020; Herby et al. 2023). Also, the Swedish emergency response was decentralized by design: the PHAS and the regions and municipalities had the primary responsibility for the pandemic response, and the government could override the PHAS only by passing a bill. Bylund and Packard (2021) note another factor: the January Agreement (*Januariavtalet*) between the government coalition and two opposition parties. We weigh this point more heavily, believing the government could have overcome the other obstacles, much as FACA requirements were overcome in the US. Koppl (2022) has noted that “constitutions can be amended, abandoned, and ignored.”

Although minority governments are common in Sweden, the government of Prime Minister Stefan Löfven was exceptionally fragile. In January 2019, a coalition composed of Löfven’s Social Democratic Party and the Green Party came into power. To ensure sufficient votes in the Parliament, the coalition struck a deal, the “January Agreement,” with the Center Party and the Liberals on 19 January 2019. It bound the government to a 73-point program that included liberal elements such as a call for “better conditions for businesses and entrepreneurs” and changes in the tax laws to benefit growing small- and medium-size enterprises. Lockdown would have been inconsistent with these principles, and Löfven’s red-green coalition was so fragile that a deviation from the Agreement would have sunk it. The final collapse of the Löfven II government illustrates this fragility. In July of 2021 the government tried to honor the Agreement’s call for rent reform by proposing a liberalization measure that would have given landlords greater freedom to increase rents. The socialist Left Party called for a vote of no confidence, and the government fell.

The January agreement bound the Löfven government to a rigid set of policies so that it could not benefit from policy discretion, a key assumption in H1. Sweden is, then, a “non-falsifying exception” (Molnar 1967, 7) to our first hypothesis. Andersson and Aylott (2020) discuss the coalition weakness as a contributing factor, but without mentioning the January Agreement.

While South Korea, a presidential republic, is not among our case studies, it, too, had an unusual policy that did not include lockdowns. Presumably, presidential politics played its part in this policy choice. Incumbent president Moon Jae-in faced an election in April 2020 and may have feared the electoral consequences of the economic harms lockdowns would cause. In the wake of Korea’s 2015 MERS outbreak, it had built up good infrastructure for test and trace, upon which the president relied in 2020. Thus, the perceived epidemiological benefit of lockdown was lower than in many other countries and (as in Poland) the political costs were higher. In any event, the government’s crucial advisory body was the powerful Central Disaster Safety and Countermeasures Headquarters (CDSCHQ), which the President “assembles” in a crisis. CDSCHQ supervises local bodies with varied responsibilities including “formulating plans” and “providing technical guidance” (Kim et al. 2021, 782). The law creating the CDSCHQ says, “The Prime Minister shall serve as the Chairperson of the Central Committee, and the heads of central administrative agencies or *related institutions or organizations prescribed by Presidential Decree* shall serve as the members of the Central Committee” (KLRI 2018 emphasis added). Because its membership can be so freely controlled by the President, the CDSCHQ is an ad hoc body. It seems to have tight control of regular government bureaus such as local bodies and the Korea Disease Control and Prevention Agency (KDCA) and, therefore, effective control of the scientific advice they generate. The discussion of Kim et al. (2022) of Korea’s earlier MERS response is illuminating in this regard. Though outside our data set, South Korea seems to fit our theory.

## Concluding remarks

Our study shows how the political interests of governments influenced the organization of public health inquiry during the Covid-19 pandemic. Policymakers generally chose ad hoc SACs over standing bodies to better control science and to steer it towards specific political objectives. In “Delanovian” crises such as the Covid-19 pandemic, governments generally prefer the *bespoke science* of an ad hoc SAC to the off-the-rack science of a standing agency. In the limiting case, policy does not follow science, but science follows policy. Despite a large scholarly literature to the contrary, it is widely believed that science is pristine and free from politics. However, as public choice scholars have noted, science is no less a human affair than selling shoes or drinking beer (Geloso and March 2021). Tullock (1966, 158) noted the potential for “the interests” to turn economics into a “racket”. He thought “there are no significant motives for attempting to obscure or conceal truth in the natural sciences” because in them the “organization of inquiry” is “a system of voluntary cooperation in which the work of each investigator not only meets his own desires but also helps other investigators” (Tullock 1966, 158). But the natural and social sciences alike cease to be fully voluntary when governments become “entangled” (Podemska-Mikluch and Wagner 2020; Wagner 2020) with them. Entanglement may permit “the interests” to impair error elimination even in the natural sciences. Public choice theory can help identify the circumstances tending to promote or thwart error elimination in science. If the nature of the error-elimination process in science matters, public choice scholars should spend more time studying the organization of inquiry in public health and all of science. As the Covid-19 pandemic attests, this line of inquiry is much needed.

## Footnotes

1 Available at the time of this writing at https://www.protezionecivile.gov.it/it/normativa/ocdpc-n--630-del-3-febbraio-2020-primi-interventi-urgenti-di-protezione-civile-in-relazione-all-emergenza-relativa-al-rischio-sanitario-connesso-all-i/.

2 https://assets.publishing.service.gov.uk/government/uploads/system/uploads/attachment_data/file/1058442/S0369_Precautionary_SAGE_meeting_on_Wuhan_Coronavirus__WN-CoV____1_.pdf

3 Act of 2 March 2020 on special measures related to the prevention, counteracting, and combating of COVID-19, other infectious diseases, and crises caused by them (also known in Poland as Specustawa koronawirusowa): https://isap.sejm.gov.pl/isap.nsf/download.xsp/WDU20200000374/T/D20200374L.pdf

4 Prof. Ewa Łętowska, "Ustawa o koronawirusie narusza konstytucję," Wyborcza.pl, published online 3 March 2020. URL: https://wyborcza.pl/7,75398,25755535,prof-ewa-letowska-ustawa-o-koronawirusie-narusza-konstytucje.html; Prof. Jerzy Zajadło, "Three sins of the special law regarding the coronavirus," oko.press, published online 4 March 2020. URL: https://oko.press/prof-zajadlo-trzy-grzechy-specustawy-dotyczacej-koronawirusa.

5 Some authors point out a negligent attitude towards the pandemic among the Law and Justice politicians, government officials, and the head of the State Sanitary and Epidemiological Inspectorate (Kalan 2020; Styczyńska and Zubek 2023). Regardless, the Polish lockdowns were implemented on the same timeline as in Western Europe and the United States (Hale et al. 2021).

6 Founding document of the Medical Council for Covid-19 (Rada Medyczna) available at: https://www.dropbox.com/scl/fi/jyqptd8mnqtrlz9oazftg/SAB-1-founding-document.docx?rlkey=8mll2hiem2j7y7d27vymos7ku&e=1&dl=0 and sourced from the Polish Government’s official COVID-19 website: https://www.gov.pl/web/koronawirus/walka-z-pandemia-covid-19--jak-to-robimy.

7 Link to the founding document: https://www.gov.pl/web/koronawirus/walka-z-pandemia-covid-19--jak-to-robimy.

8 Ojczyk, Jolanta. (2022). "Premier powołał nową radę do spraw Covid-19." *Prawo.pl.* Published online 9 February 2022. Available at: https://www.prawo.pl/zdrowie/nowa-rada-do-spraw-covid-19,513367.html.
